# Distribution of Radionuclides and Radiological Health Assessment in Seih-Sidri Area, Southwestern Sinai

**DOI:** 10.3390/ijerph191710717

**Published:** 2022-08-28

**Authors:** Gharam A. Alharshan, Mohamed S. Kamar, El Saeed R. Lasheen, Antoaneta Ene, Mohamed A. M. Uosif, Hamdy A. Awad, Shams A. M. Issa, Hesham M. H. Zakaly

**Affiliations:** 1 Physics Department, College of Science, Princess Nourah Bint Abdulrahman University, P.O. Box 84428, Riyadh 11671, Saudi Arabia; 2 Nuclear Materials Authority, P.O. Box 530, El Maadi, Cairo 11728, Egypt; 3 Geology Department, Faculty of Science, Al-Azhar University, Cairo 11884, Egypt; 4 INPOLDE Research Center, Faculty of Sciences and Environment, Department of Chemistry, Physics and Environment, Dunarea de Jos University of Galati, 47 Domneasca Street, 800008 Galati, Romania; 5 Physics Department, College of Science, Jouf University, Sakaka 72388, Saudi Arabia; 6 Geology Department, Faculty of Science, Al-Azhar University, Assiut Branch, Cairo 71524, Egypt; 7 Physics Department, Faculty of Science, Al-Azhar University, Assiut Branch, Cairo 71524, Egypt; 8 Physics Department, Faculty of Science, University of Tabuk, Tabuk 71451, Saudi Arabia; 9 Institute of Physics and Technology, Ural Federal University, 620002 Yekaterinburg, Russia

**Keywords:** radioactivity, radiological hazard indices, Seih-Sidri area, Egypt

## Abstract

The current contribution goal is to measure the distribution of the radionuclide within the exposed rock units of southwestern Sinai, Seih-Sidri area, and assess the radiological risk. Gneisses, older granites, younger gabbro, younger granites, and post granitic dikes (pegmatites) are the main rock units copout in the target area. Radioactivity, as well as radiological implications, were investigated for forty-three samples from gneisses (seven hornblende biotite gneiss and seven biotite gneiss), older granites (fourteen samples), and younger granites (fifteen samples of syenogranites) using NaI (Tl) scintillation detector. External and internal hazard index (H_ex_, H_in_), internal and external level indices (Iα, Iγ), absorbed dose rates in the air (D), the annual effective dose equivalent (AED), radium equivalent activity (Ra_eq_), annual gonadal dose (AGDE), excess lifetime cancer risk (ELCR), and the value of Upper Continental Core ^232^Th/^238^U mass fractions were determined from the obtained values of ^238^U, ^232^Th and ^40^K for the examined rocks of Seih-Sidri area. The average ^238^U mg/kg in hornblende biotite gneiss and biotite gneiss, older granites, and syenogranites is 2.3, 2.1, 2.7, and 8.4 mg/kg, respectively, reflecting a relatively higher concentration of uranium content in syenogranites. The results suggest that using these materials may pose risks to one’s radiological health.

## 1. Introduction

In health physics, natural radioactivity research is particularly important in terms of determining the radiation exposure to humans from natural radiation and for other practical reasons. As a result, several studies have been undertaken across the globe to determine natural radiation levels [1,2,3,4,5,6,7,8,9]. Because these radionuclides are found everywhere on the world’s surface, such as soils, rocks, plants, water, air, and construction materials, including the human body, ^238^U, ^232^Th, and ^40^K radionuclides are a persistent and unavoidable characteristic of life on earth [10]. Levels of mg/kg of ^238^U and ^232^Th series decay products have been found in the Earth’s crust [11,12]. ^40^K radioisotope is a single natural radionuclide that makes up 0.0118 percent of total potassium in the Earth’s crust. The mass fraction of ^238^U, ^232^Th, and ^40^K in the soil of the Earth varies from place to place since their levels are dependent on the origins of the soil and the kind of rocks [13]. Natural radioactivity is based on geological environments, especially special rock types and various processes that cause fractionations [14]. It is also possible that the area of investigation considered is one of the most important areas in southwest Sinai. In many studies, different authors have used geospatial techniques as structural techniques, seismology and geophysics as well as Safe Urban Extension Studies in southwestern Sinai [15,16,17]. Due to the limited uranium mineralization and the accompanying radiation exposure zones, it is possible that the predicted impacts on the ecosystem will be minimal.

Crystalline rocks of Egypt, which are dominantly distributed in the Eastern Desert and South Sinai as well as the Uwainate area, constitute the northern sector of the Arabian Nubian Shield (ANS) [18,19,20]. These rocks include infrastructure gneisses, ophiolite and arc assemblages, and variable compositions of granitic intrusion [21,22,23,24]. ANS represents the main crustal juvenile that developed during the late Proterozoic Era by closing the Mozambique Ocean [19,21], exposed from Egypt to Ethiopia on the Nubian side and from Saudi Arabia to Yemen on Arabian Side [25]. Syn-, late- and post-orogenic granitic rocks of Late Cryogenian–Ediacaran are widely distributed in ANS, covering about sixty percent of the Egyptian Neoproterozoic rocks [26]. The Eastern Desert of Egypt’s Side may be divided primarily into the North, Central, and Southeastern Deserts [25]. While gneiss and ophiolitic assemblages are prevalent in the central and southern sectors of the Egyptian Eastern Desert, granites are extensively dispersed in the region’s northern region [19,27]. Late Cryogenian–Ediacaran granitic intrusions have a different age, mineralogical, and geochemical composition, as well as the tectonic regime. Syn-orogenic granitic rocks are the oldest ones (850–610 Ma) covering about twenty-seven percent of the Egyptian basement rocks with the composition varying from tonalite to granodiorites. On the other hand, the youngest ones (610–550 Ma) represent late- to post-orogenic granites with I- to A-granitic types.

The major goal of this research is to look at the natural radioelement (^238^U, ^226^Ra, ^232^Th, and ^40^K) distributions in variable rock units in the Seih-Sidri area, southwestern Sinai, as well as their radiological consequences. The radiological hazards associated with these rocks were calculated including external and internal hazard indices, activity index, absorbed dose rates in air, external and internal level indices, radium equivalent activity, and annual effective dose. Other metrics covered include the annual gonadal dose (AGED) for a house inhabitant, the excess lifetime cancer risk (ELCR), and the Upper Continental Crust (UCC) value ^232^Th/^238^U mass fraction [28,29].

## 2. Materials and Methods

### 2.1. Sample Processing

A total of 43 samples were collected in Seih-Sidri area from several rock types, as follows: 7 hornblende biotite gneisses (HBG1–HBG7); 7 biotite gneisses (HG1–HG7); 14 older granites (OG1–OG14); and 15 syenogranites (Sy1–Sy15). The mass fraction of uranium (mg/kg), thorium (mg/kg), radium (mg/kg) and potassium (%) in different rock types were detected γ-spectrometric by using a multi-channel analyzer with a 76 × 76 mm NaI (Tl) scintillation detector. The studied rocks were crushed and ground (1mm grain size) and then put in a plastic cylindrical beaker (212.6 cm^3^), which has the same diameter as the detector (76 mm). These containers were charged with 300–400 g of sample and sealed well, then left for at least 28 days to accumulate free radon and other isotopes emissions. The standards were measured the first two times, each time about 100 s. The average of the total count for each sample was determined and divided by its net weight, and then introduced into computer program analysis, whereas the mass fractions of U, Th, (Ra), and K% were calculated.

### 2.2. Activity Measurements (NaI (Tl) Detector)

Despite the NaI (Tl) detector having a low energy resolution, it has good efficiency. Its high efficiency allows it to determine ^238^U, ^40^K, and ^232^Th mass fractions in rock samples quickly and precisely. After sample preparation operations, they were subjected to gamma assay according to the following steps:

1. Equipment processing using reference gamma emission sources (^137^Cs and ^57^Cs) for lead energy calibration shield.
2. Test samples for 1000 s for each one, in a protected environment and design the total numbers of U, Th, eU (Ra), and K they have Selected energy zones as well.
3. The background spectra were used to modify the net peak area of gamma rays for the measured isotopes. Background count rates in specific energy regions (ROIs) for the laboratory with a detector. The assessment time for activity or background was the same.
4. Computer “analysis” Canberra software was used to process recorded spectral data (total number of U, Th, Ra, and K) for each sample, determining the U, Th, and Ra mass fractions in mg/kg and the potassium mass fraction in percentage using background count rates, sample weight, measurement time, and initial sensitivity constants (percent). For low and medium-grade samples, the lowest detection limit for U is 2 mg/kg, while the maximum one is 2000 mg/kg. Limits of detection for raw granite samples exceed 2 percent. Uranium-specific activity uncertainty is between 10 and 15 percent. The inaccuracy is anticipated to range from 1 to 5 percent, while the minimum detection level for thorium is 0.6 mg/kg. Radium detection threshold of 0.4 mg/kg. The Ra estimated error percentages range from 1 to 5 percent. The precision of the energy calibration approach, which takes into account the likely interference of each nuclide in each peak site, and the estimated error of 1 to 5 decide the findings [30,31,32,33]. The minimal detection K is 0.1 percent. The gamma-ray spectrometry system consists of a scintillation (Bicron) detector with a 76 mm × 76 mm NaI (Tl) crystal that is hermetically sealed with a photomultiplier tube in an aluminum container. The detector is fastened to the “Accuspec card” and covered with its amplifier in a cylindrical lead chamber and a shield of copper with a thickness of 0.6 cm against induced X-rays. The Accuspec NaI detector with a 2K onboard ADC, Amp, and HVPS with a 2K channel memory is linked to a PC. By choosing four energy regions of interest (ROIs) for U, Th, Ra, and K, which correspond to ^234^Th, ^212^Pb, ^214^Pb, and ^40^K, respectively, the radionuclides are measured. Thorium is measured in eTh, whereas uranium is measured in U and Ra (U). The U values estimate the first daughter isotope in the ^238^U decay chain with the least amount of loss by utilizing the ^234^Th energy peak (93 keV) to reflect the mass fraction of U. When ^238^U and all of its daughter isotopes reach secular equilibrium, which takes place when the daughters’ rate of decay equals the parent’s one after four weeks, radium is measured at the ^214^Pb peak of 352 keV energy, which is tied to a measure for the mass fraction of the U. Thorium is found in the Pb-212 peak, which has an energy of 238 keV [31,34,35].

### 2.3. Energy and Efficiency Calibration

Changes in the power supply, voltage, and photomultiplier tube amplification properties affect how stable the energy of the spectrometer channels is. The temperature affects both the power source and the photomultiplier’s properties. Using radioactive calibration sources such as ^137^C (produced in channel 662) and ^57^Co (122.1 keV, prepared in channel 122), a permanent calibration is carried out to guarantee that the instrument reliably records the gamma radiation energy of the radioactive elements: (I) gain adjustments are made first with the ^137^Cs source before zero changes are made with the ^57^Co source, and (II) the ^137^Cs source is utilized frequently as a minimum method.

By using standard sources (IAEA-314) having specific activity for ^226^Ra, (732 Bqkg^−1^) and ^232^Th (17.8 ppm), the efficiencies have been calculated experimentally. Theoretically, in a program built in MATLAB, the absolute efficiency at any interesting γ–energy in the energy ranged from 10 to 1764 keV [2].

For quality assurance, the uncertainty of activity *u*(*A*) was evaluated using the following formula $u\left(A\right)=A\sqrt{[\frac{u\left(N_{p}\right)}{N_{p}}{]}^{2}+[\frac{u\left(\eta \right)}{\eta }{]}^{2}+[\frac{u\left(m\right)}{m}{]}^{2}+[\frac{u\left(P\gamma \right)}{P\gamma }{]}^{2}}$ [36,37] where the uncertainty of net count rate, absolute efficiency for each gamma line discovered in the same number of channels in the sample, the mass of the sample, and absolute transition probability of decay are represented by *u*(*N_p_*), *u*(*η*), *u*(*m*), and *u*(*Pγ*), respectively. The uncertainty of each unique net-peak area was estimated by computer “analysis” software.

## 3. Results and Discussion

### 3.1. Geological Setting

**Figure 1:**
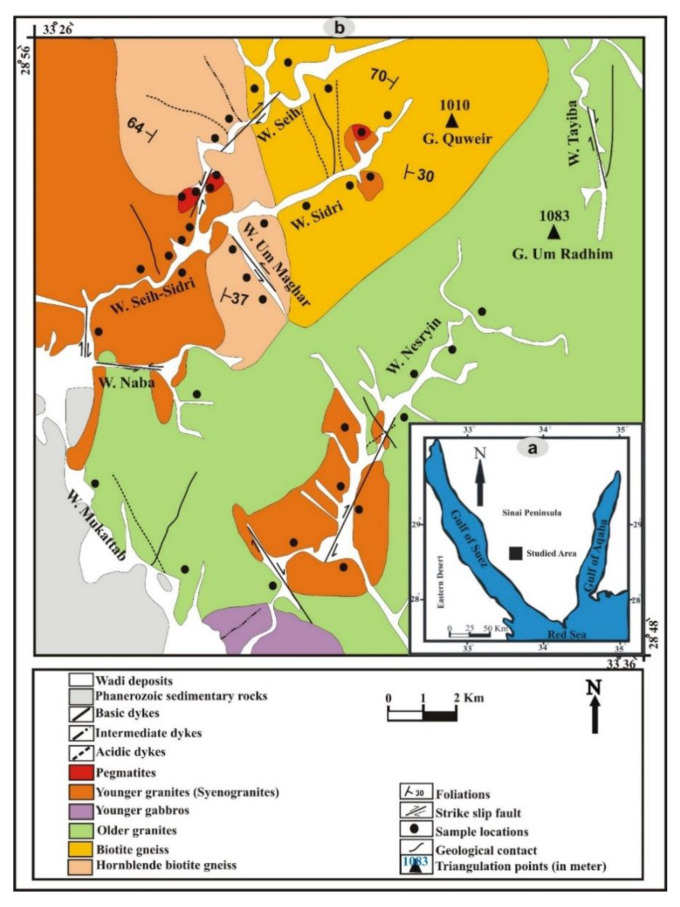
(**a**) Geographic map of Sinai, and (**b**) detailed geological map of Seih-Sidri area, southwestern Sinai, Egypt [38].

Seih-Sidri area is located in southwestern Sinai (latitudes 28°48′ and 28°55′ N and longitude 33°26′ to 33°36′ E) [38,39,40] (Figure 1a). The tectono-stratigraphic sequence of the study area can be arranged according to the field observations and relationships between the different rock varieties from the oldest to youngest as follows: 1-Gneisses, 2-Older granites, 3-Younger gabbros, 4-Younger granites, 5-Pegmatites, and 6-Post granite dykes (Figure 1b).

Gneisses occupy large areas (102 km^2^) and are exposed at Wadi Seih, Wadi Um Maghar, and Wadi Sidri. They are medium- to coarse-grained with greyish color. Gneisses form moderate relief and are highly weathered, jointed, and dissected by strike-slip fault. They are cut by acidic, intermediate, and basic dykes and sometimes show bedding planes and well-developed foliation. Gneisses comprise hornblende biotite-gneiss and biotite gneiss. Older granites from moderate to high topographic relief terrains cover an area of about 202 km^2^. The rocks are exposed in Wadis Nisryin, Mukattab, Naba, and Tayiba. They are generally hard massive, dark greenish-grey in color, and medium- to coarse-grained. The rocks show exfoliation and block weathering and are highly jointing. They intrude gneisses and were intruded by younger gabbro and younger granites. The rocks are cut by acidic, intermediate, and basic dykes. They are represented by quartz diorite and granodiorite with gradational contact.

**Figure 2:**
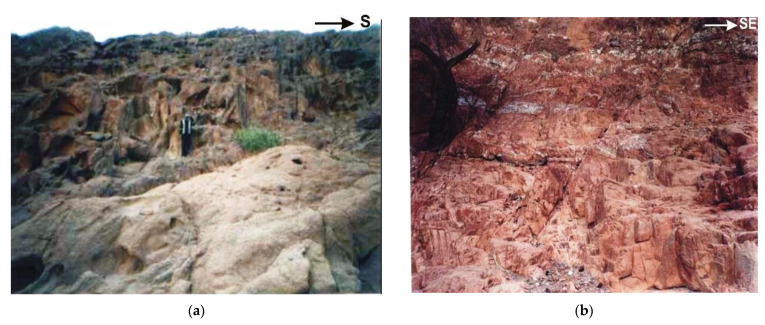
Field photographs reveal (**a**) cavernous weathering of syenogranite at W. Seih-Sidri; and (**b**) pegmatites at W. Sidri, southwestern Sinai, Egypt.

Younger gabbro covers about 3.7 km^2^ at the entrance of Wadi Nisryin. It is dark green to greyish-green, medium to coarse-grained, sometimes the rocks occur as roof pendants uplifted by the younger granites. It forms an arcuate body with moderate to high relief. Younger granites cover about 37 km^2^ and are exposed at Wadis Nisryin, Naba, Seih-Sidri, Teima, and Seih and the mouth of Wadi Sidri. It is medium- to coarse-grained with pink color and attains medium to high relief. They are marked by cavernous weathering and highly jointing (Figure 2a). Many of these joints are filled by copper mineralization or by pegmatite bodies which contain noticeable high radioactivity.

Younger granites intrude all the previously mentioned rock units and have sharp contact with the surrounding rocks. Pegmatites are very coarse-grained (Figure 2b) and can be classified according to field observation into two types: zoned pegmatite and unzoned pegmatite. Post granite dykes occur as single dyke or form swarms cutting all the rock units. They are classified into acidic, intermediate, and basic dykes. Porphyritic dacite, porphyritic rhyodacite, and rhyolite rocks represent acidic dykes. While the intermediate dykes are represented by andesite rock and the basic dykes are represented by doleritic and basaltic rock variety. The relative age relation of the dykes indicates that the acidic dykes are the oldest, followed by intermediate and the basic as the youngest one.

### 3.2. Radioactivity and Radiometric Prospecting

The radioactivity concentrations of NORMs for 43 several rock types (7 hornblende biotite gneisses; 7 biotite gneisses; 14 older granites; and 15 syenogranites) in the Seih-Sidri area in Egypt were measured using gamma-ray spectrometry. To estimate the potential radiation risks due to the utilization of these rock types, the different risk indexes and annual effective doses were also evaluated. Our results were compared with the global average values established by UNSCEAR. Our results and comparisons are presented systematically in the following subsections.

**Figure 3:**
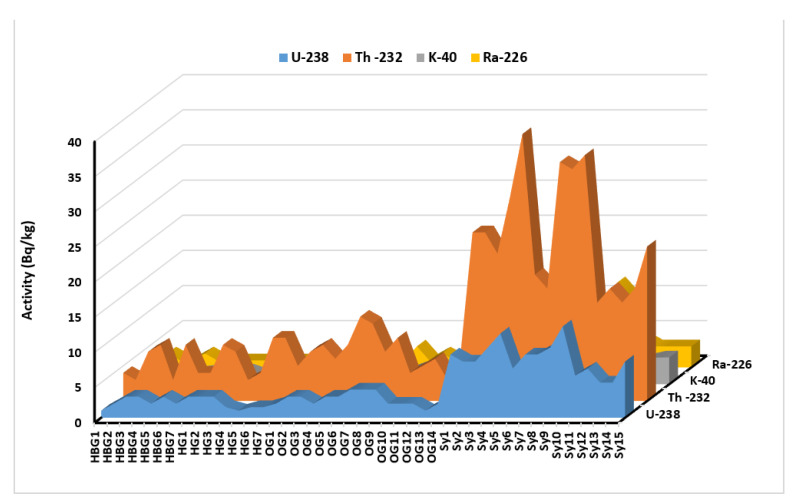
Representation of the radiometric measurements of ^238^U, ^226^Ra, ^232^Th, and ^40^K (ppm) in the samples of Seih-Sidri area, Egypt.

The average, lowest, and maximum values of the dry weight activity concentrations of ^238^U, ^232^Th, and ^40^K for the rock types examined in the current investigation are shown in Figure 3 and summarised in Table 1. The average activities of the daughter radionuclides ^214^Pb and ^214^Bi were used to estimate the concentrations of ^238^U activity, while the concentrations of ^212^Pb, ^208^Tl, and ^228^Ac were used to estimate the concentrations of ^232^Th activity. The concentration of ^40^K in various samples was assessed using the 1461 keV gamma activity. For each sample and isotope under investigation, a variety of actions are seen. The presence of radioactive minerals and a rock’s capacity to absorb certain elements may explain differences in the concentration of NORM activity among different types of rock.

The current study showed that the distribution of uranium and thorium in the study rocks increases from basic to acidic types [35]. This trend may be attributed to the fact that in the early stages of magmatic evolution the principal minerals were pyroxenes, amphiboles, and plagioclase. The small and low-charged ions in the mafic minerals do not permit the entrance of large and highly charged ions of U and Th in their lattices, so the abundance of incompatible elements U and Th in mafic rocks is low [34]. In the late stage of the magmatic evolution, the principal minerals are feldspars, biotite, and muscovite with some accessory minerals such as allanite, apatite, monazite, and zircon. All these minerals permit the entrance of U and Th in their lattices [34]. The average mass fraction of uranium in hornblende biotite gneiss is 28.2 Bq/kg, while in biotite gneiss is 25.6 Bq/kg. Thorium average contents in hornblende biotite gneiss is 21.5 Bq/kg, while in biotite gneiss is 25.5 Bq/kg.

The Th/U ratios are 2.38 and 3.47 in hornblende biotite gneiss and biotite gneiss, respectively. The U mass fraction in older granites has an average of 2.64 mg/kg. The Th mass fractions have an average of 6.93 mg/kg. The average of Th/U = 2.67 reflects enrichment in Th and depletion of U. The studied syenogranite has a U range between 5 and 13 mg/kg with an average of 8.40 mg/kg and the Th mass fractions range between 14 and 38 mg/kg with an average of 23.60 mg/kg. The studied syenogranites have U and Th mass fractions higher than the Upper Continental Crust value, therefore it pertained to uraniferous syenogranite. The average Th/U ratio of the study syenogranite is 2.79 mg/kg as shown in Table 1 and Figure 3.

**Figure 4:**
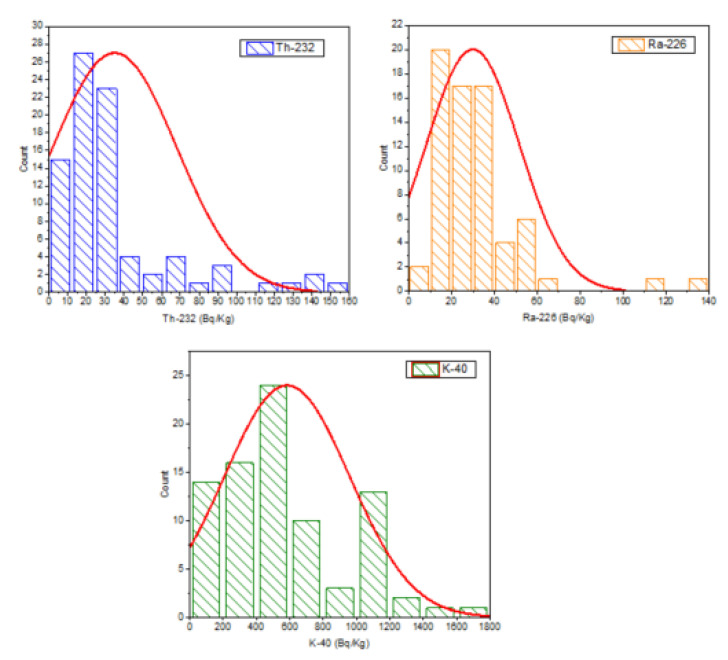
Frequency distribution of the ^232^Th, ^226^Ra, and ^40^K, respectively, in the, studied rock samples.

In Figure 4, the histograms are presented for the data of the activity concentration results (in Bq/kg) for ^238^U, ^232^Th, ^226^Ra, and ^40^K in forty-three different samples of the studied rocks. The data present normal distribution from this figure.

Figure 4 shows the histograms for the findings of the activity concentrations for ^238^U, ^232^Th, ^226^Ra, and ^40^K in distinct samples of the investigated rocks (in Bq/kg). From this graph, the data show a clear distribution, and all activity concentration levels are almost within the estimated mean value for each natural radionuclide.

Several indices were developed based on the mass fractions found in this work for ^40^K (C_K_), ^232^Th (C_Th_), and ^226^Ra (C_Ra_) to quantify the radiological danger for people exposed to the radiations generated by the granite rocks [10,32,33,34]. The absorbed dose rate in the air (D) could be calculated using the following formula: D (nGy h^−1^) = 0.0417 C_K_ + 0.604 C_Th_ + 0.462 C_Ra_ [33]. The following equation gives the external hazard index (Hex), which is frequently used to assess the radiation dose rate brought on by external exposure to gamma radiation from natural radionuclides in soil/rock samples: H_ex_ is equal to [33] = C_K_ 4810 + C_Th_ 259 + C_Ra_ 370 ≤ 1. For the radiation threat to be negligible, the estimated average external hazard index must be less than unity.

The internal hazard index regulates the internal exposure to ^222^Rn and its radioactive offspring (H_in_). H_in_ is a metric used to calculate the harmful effects of radioactive elements on the lungs and other respiratory organs. For example, the H_in_ equation can be used to quantify the risk of internal exposure to the natural radionuclides ^40^K, ^226^Ra, and ^232^Th: H_in_ = C_K_ 4810 + C_Th_ 259 + C_Ra_ 185.

**Figure 5:**
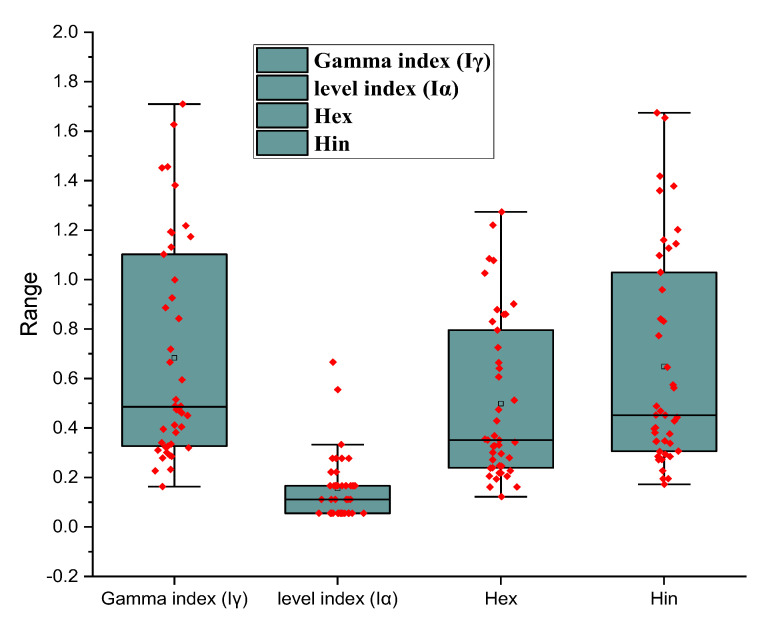
Range, mean, and median line of some radiological hazards in the measured samples.

To maintain a low radiation hazard, the external hazard index (H_ex_) must also be smaller than unity. As a result, the calculated external danger index for the analyzed samples was lower than the safety limit. The statistical distribution of the results for gamma index (Iγ), level index (Iα), external (H_ex_), and internal (H_in_) hazard index, was obtained based on the activity concentration results for ^238^U, ^232^Th, ^226^Ra, and ^40^K in the studied points of examined area are presented in Figure 5 using boxplots. The ^40^K has the highest values of the range, median, mean, and outliers of hazard indices (H_ex_ and H_in_). The high activity of potassium concentration in these rock types (7 hornblende biotite gneisses; 7 biotite gneisses; 14 older granites; and 15 syenogranites) may be due to the rocks’ efficiency in retaining potassium from the environment.

The evaluation of the gamma-ray index (Iγ), which is closely connected to the yearly effective dosage, was recommended by the European Commission [41]. The gamma-ray index is determined using a typical room model with dimensions of 4 m × 5 m × 2.8 m and walls that are 20 cm thick. Iγ = C_Ra_ 300 + C_Th_ 200 + C_K_ 3000 is the formula used to compute the index factor related to the external exposure. The following relation is used to determine the extra alpha radiation caused by radon inhalation from building materials: Iα = C_Ra_ 200 ≤ 1. I should be less than Iα = 1, which equates to 200 Bq kg^−1^ as the upper limit permitted [33]. It is estimated that a building material with a Ra content of less than 200 Bq kg^−1^ will not produce indoor radon levels of more than 200 Bq m^3^ for alpha radiation. The internal hazard index (H_in_) for the investigated rocks varied from 0.3 to 0.4 on average. These values are below the limit safety value of 1 as stated by [41] except for syanogranite samples, which had H_in_ of 1.2, a value more than the safety limit. The external hazard index (H_ex_) must also be less than unity to keep the radiation risk minimal. Because of this, the computed external risk index for the samples that were evaluated was below the permissible level. In Figure 5, boxplots are used to display the statistical distribution of the results for the gamma index (Iγ), level index (Iα), external (H_ex_), and internal (H_in_) hazard index, which were obtained based on the activity concentration results for ^238^U, ^232^Th, ^226^Ra, and ^40^K in the studied points of the examined area. The 40K has the greatest values for the hazard indices’ range, median, mean, and outliers (H_ex_ and H_in_). The ability of these rock types to effectively retain potassium from the environment may account for the high activity of potassium concentration in these rock types (7 hornblende biotite gneisses; 7 biotite gneisses; 14 older granites; and 15 syenogranites).

**Figure 6:**
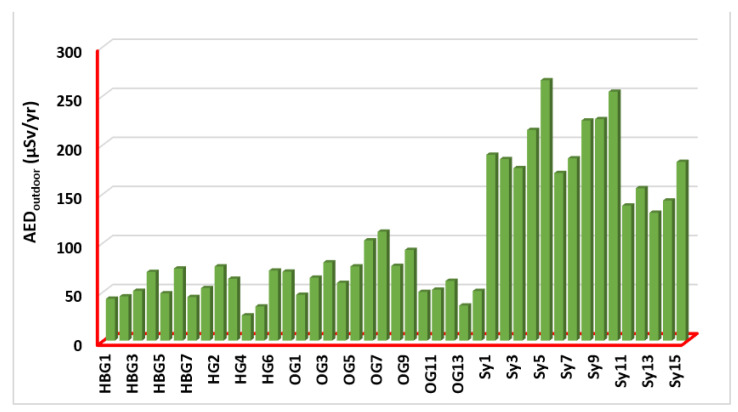
Outdoor annual effective doses (AED_outdoor_) for all (7 hornblende biotite gneisses; 7 biotite gneisses; 14 older granites; and 15 syenogranites) rock samples.

**Figure 7:**
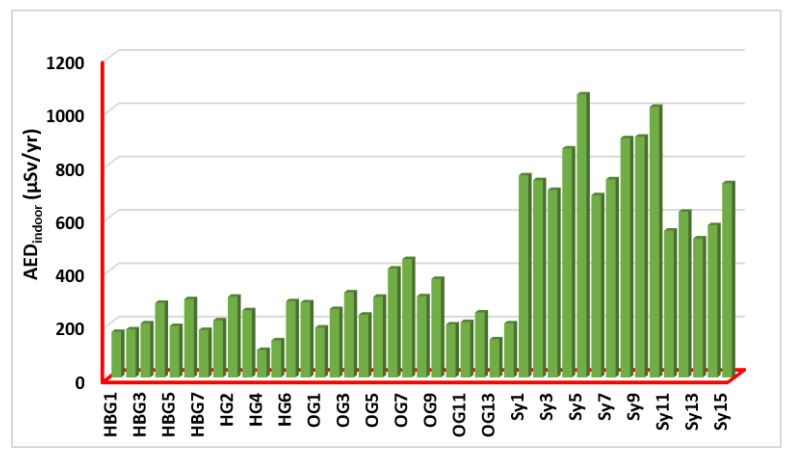
Indoor annual effective doses (AED_outdoor_) for all (7 hornblende biotite gneisses; 7 biotite gneisses; 14 older granites; and 15 syenogranites) rock samples.

The measured outdoor annual effective dose (AED_outdoor_) values for the investigated samples have been presented in Figure 6 and computed by: $AED_{outdoor}=D\left(\mathrm{nGy}\;h^{-1}\right)\times 0.2\times 24\;\left(h\right)\times 365\;\left(d\right)\times 0.7\times 10^{-6}\;\left(\mathrm{Sv}\;Gy^{-1}\right)\;$ [42,43] where 0.2 is the outdoor occupancy factor [34]; 0.7 $\left[\frac{\mathrm{Sv}}{\mathrm{Gy}}\right]$ is the conversion coefficient from the absorbed dose in the air to the effective dose received by adults; and $10^{-6}$ is the conversion factor between nano- and millimeter measurements. $D\left[\frac{\mathrm{nGy}}{h}\right]$ is the total air absorbed dose rate outdoors, and 8760 h is the number of hours in a year ($\left[\frac{24\;h\;}{\mathrm{day}}\right]$ × 365 days). The values ranged from 25.66 (HG4) to 265.30 (Sy5) μSvy^−1^, with an average value of 106.09 μSvy^−1^. That is HG4 (Biotite Gneisses) and Sy5 (Syenogranites) rocks have the lowest and highest AED_outdoor_ among all (7 hornblende biotite gneisses; 7 biotite gneisses; 14 older granites; and 15 syenogranites) samples, respectively (Figure 6). The AED_outdoor_ values are higher than the corresponding global value of 0.07 mSv. The measured indoor annual effective dose (AED_indoor_) values for the examined rock samples have been shown in Figure 7. The values are ranging from 102.60 (HG4) to 1061.19 (Sy5) μSv/yr, with an average value of 424.35 μSv/yr. HG4 and Sy5 rock samples have the lowest and highest AED_indoor_ values, respectively, among all samples (Figure 7). The AED_indoor_ values are higher than the corresponding global value of 0.410 mSv.

**Figure 8:**
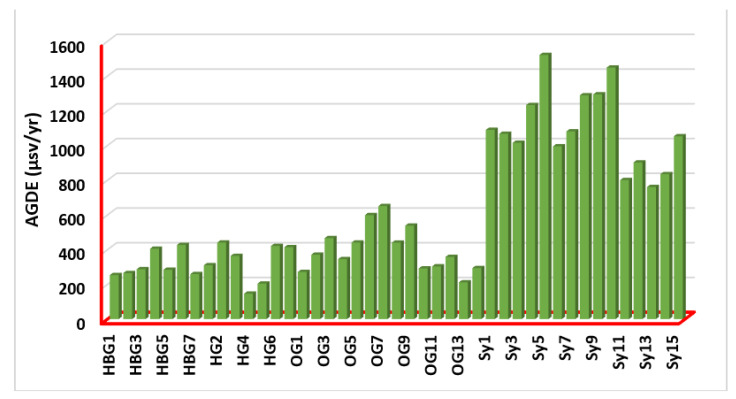
The annual gonadal equivalent dose (AGDE) in the (7 hornblende biotite gneisses; 7 biotite gneisses; 14 older granites; and 15 syenogranites) rock samples.

The annual gonadal dose equivalent (AGDE) for (7 hornblende biotite gneisses; 7 biotite gneisses; 14 older granites; and 15 syenogranites) rock samples is shown in Figure 8. The amount of AGDE produced in soil by the activity of ^226^Ra, ^232^Th and ^40^K is calculated as follows: $AGDE\;\left(\mu \mathrm{Sv}\;yr^{-1}\right)=\frac{309}{100}\times C_{Ra}+\frac{418}{100}\times C_{Th}+\frac{314}{1000}\times C_{K}\;$ [44,45]. AGDE values ranged from 146.48 (HG4) to 1521.51 (Sy5) µSv yr^−1^, with an average value of 615.19 µSv yr^−1^. The average value is much higher than its corresponding global value of 300 µSv yr^−1^. These measurements supply information on the four different types of rocks (hornblende biotite gneisses, biotite gneisses, older granites, and syenogranites) that these models require to construct guidelines about radiological health care. According to the findings, there are potential dangers to one’s radiological health associated with making use of these materials.

## 4. Conclusions

Radiological impact and radionuclides distribution within gneisses, older granites, syenogranite of Seih-Sidri area, southwestern Sinai, Egypt using γ-ray (NaI) spectrometry technique. For the examined rocks, the average internal hazard index (H_in_) ranged from 0.3 to 0.4. Except for syanogranite samples, which had H_in_ of 1.2, greater than the safety limit, these values are below the limit safety value of 1, as suggested by the European Commission (1999). To maintain a low radiation hazard, the external hazard index (H_ex_) must also be smaller than unity. As a result, the calculated external danger index for the examined samples was lower than the safety limit, suggesting that they can be used in industrial applications. These measurements offer data on the four different types of rocks (hornblende biotite gneisses, biotite gneisses, older granites, and syenogranites) that these models need to develop radiological health care standards. According to the findings, using these samples poses a risk to radiological health. On the other hand, syenogranite samples pose a risk to radiological health according to their high internal hazard (Hin).

## Figures and Tables

**Table 1:** Average, minimum (Min), and maximum (Max) radiometric measurements of the studied rocks in the study area.

Rock Units	Activity	^238^U (mg/kg)	^232^Th (mg/kg)	^226^Ra (mg/kg)	^40^K (%)	^232^Th/^238^U
Hornblende Biotite Gneisses	Average	2.3	5.3	1.3	1.4	2.4
	Min	1.0	3.0	1.0	0.6	1.5
	Max	3.0	8.0	2.0	1.8	4.0
Biotite Gneisses	Average	2.1	6.3	1.3	1.5	3.5
	Min	1.0	3.0	1.0	0.4	1.3
	Max	3.0	9.0	3.0	2.2	6.0
Older Granites	Average	2.6	6.9	2.0	1.8	2.7
	Min	1.0	3.0	1.0	1.1	1.8
	Max	4.0	12.0	3.0	3.2	4.0
Syenogranites	Average	8.4	23.6	5.0	3.8	2.8
	Min	5.0	14.0	3.0	3.4	1.8
	Max	13.0	38.0	12.0	4.7	3.8

## Data Availability

The data presented in this study are available on request from the corresponding authors.

## References

[1] Zare M.R., Mostajaboddavati M., Kamali M., Abdi M.R., Mortazavi M.S. (2012). 235U, 238U, 232Th, 40K and 137Cs Activity Concentrations in Marine Sediments along the Northern Coast of Oman Sea Using High-Resolution Gamma-Ray Spectrometry. Mar. Pollut. Bull..

[2] Zakaly H.M.H., Uosif M.A., Issa S., Saif M., Tammam M., El-Taher A. (2019). Estimate the Absolute Efficiency by MATLAB for the NaI (Tl) Detector Using IAEA-314. AIP Conf. Proc..

[3] Zaim N., Tugrul A.B., Atlas H., Buyuk B., Demir E., Baydogan N., Altinsoy N. (2016). Investigation of Natural Radioactivity of Surface Soil Samples in the Vicinity of Edirne-Turkey. Acta Phys. Polonica A.

[4] Ribeiro F.C.A., Silva J.I.R., Lima E.S.A., do Amaral Sobrinho N.M.B., Perez D.V., Lauria D.C. (2018). Natural Radioactivity in Soils of the State of Rio de Janeiro (Brazil): Radiological Characterization and Relationships to Geological Formation, Soil Types and Soil Properties. J. Environ. Radioact..

[5] Öztürk B.C., Çam N.F., Yaprak G. (2013). Reference Levels of Natural Radioactivity and 137Cs in and around the Surface Soils of Kestanbol Pluton in Ezine Region of Çanakkale Province, Turkey. J. Environ. Sci. Health Part A Toxic/Hazard. Subst. Environ. Eng..

[6] Zakaly H.M.H., Uosif M.A.M., Issa S.A.M., Tekin H.O., Madkour H., Tammam M., El-Taher A., Alharshan G.A., Mostafa M.Y.A. (2021). An extended assessment of natural radioactivity in the sediments of the mid-region of the Egyptian Red Sea coast. Mar. Pollut. Bull..

[7] Günay O., Eke C. (2019). Determination of Terrestrial Radiation Level and Radiological Parameters of Soil Samples from Sariyer-Istanbul in Turkey. Arab. J. Geosci..

[8] Aközcan S. (2014). Annual Effective Dose of Naturally Occurring Radionuclides in Soil and Sediment. Toxicol. Environ. Chem..

[9] El-Taher A., Zakaly H.M.H., Elsaman R. (2018). Environmental Implications and Spatial Distribution of Natural Radionuclides and Heavy Metals in Sediments from Four Harbours in the Egyptian Red Sea Coast. Appl. Radiat. Isot..

[10] UNSCEAR (2010). Sources and Effects of Ionizing Radiation United Nations Scientific Committee on the Effects of Atomic Radiation.

[11] Abbasi A., Mirekhtiary F. (2020). Heavy Metals and Natural Radioactivity Concentration in Sediments of the Mediterranean Sea Coast. Mar. Pollut. Bull..

[12] Abbasi A., Zakaly H.M.H., Mirekhtiary F. (2020). Baseline levels of natural radionuclides concentration in sediments East coastline of North Cyprus. Mar. Pollut. Bull..

[13] El Rakaiby M.L., Shalaby M.H. (1992). Geology of Gebel Qattar Batholith, Central Eastern Desert, Egypt. Int. J. Remote Sens..

[14] Heikal M.T.S., Top G. (2018). Assessment of Radioactivity Levels and Potential Radiation Health Hazards of Madsus Granites and Associated Dikes Nearby and around Ruwisat Village, South Sinai, Egypt. J. Afr. Earth Sci..

[15] Ramadan R.S., Dawood Y.H., Yehia M.M., Gad A. (2022). Environmental and Health Impact of Current Uranium Mining Activities in Southwestern Sinai, Egypt. Environ. Earth Sci..

[16] Omar A., Arnous M., El-Ghawaby M., Alshami A., El Zalaky M. (2016). Seismotectonic hazards assessment in Southwestern Sinai area using Remote sensing and GIS. Sinai J. Appl. Sci..

[17] Sultan Y.M., El-Shafei M.K., Arnous M.O. (2017). Tectonic Evolution of Kid Metamorphic Complex and the Recognition of Najd Fault System in South East Sinai, Egypt. Int. J. Earth Sci..

[18] Awad H.A., El-Leil I.A., Nastavkin A.V., Tolba A., Kamel M., El-Wardany R.M., Rabie A., Ene A., Tekin H.O., Issa S.A.M. (2022). Statistical Analysis on the Radiological Assessment and Geochemical Studies of Granite Rocks in the North of Um Taghir Area, Eastern Desert, Egypt. Open Chem..

[19] Lasheen E.S.R., Saleh G.M., Khaleal F.M., Alwetaishi M. (2021). Petrogenesis of Neoproterozoic Ultramafic Rocks, Wadi Ibib–Wadi Shani, South Eastern Desert, Egypt: Constraints from Whole Rock and Mineral Chemistry. Appl. Sci..

[20] Hamdy M.M., Lasheen E.S.R., Abdelwahab W. (2022). Gold-Bearing Listwaenites in Ophiolitic Ultramafics from the Eastern Desert of Egypt: Subduction Zone-Related Alteration of Neoproterozoic Mantle?. J. Afr. Earth Sci..

[21] Saleh G.M., Khaleal F.M., Lasheen E.S.R. (2022). Geochemistry and Paleoweathering of Metasediments and Pyrite-Bearing Quartzite during the Neoproterozoic Era, Wadi Ibib-Wadi Suwawrib, South Eastern Desert, Egypt. Arab. J. Geosci..

[22] Alzahrani A.M., Lasheen E.S.R., Rashwan M.A. (2022). Relationship of Mineralogical Composition to Thermal Expansion, Spectral Reflectance, and Physico-Mechanical Aspects of Commercial Ornamental Granitic Rocks. Materials.

[23] Kamar M.S., Salem I.A., El-Aassy I.E., El-Sayed A.A., Awad H.A., Tekin H.O., Alzahrai A.M., Lasheen E.S.R. (2022). Petrology and Geochemistry of Multiphase Post-Granitic Dikes: A Case Study from the Gabal Serbal Area, Southwestern Sinai, Egypt. Open Chem..

[24] Khaleal F.M., Saleh G.M., Lasheen E.S.R., Alzahrani A.M., Kamh S.Z. (2022). Exploration and Petrogenesis of Corundum-Bearing Pegmatites: A Case Study in Migif-Hafafit Area, Egypt. Front. Earth Sci..

[25] Stern R.J., Hedge C.E. (1985). Geochronologic and Isotopic Constraints on Late Precambrian Crustal Evolution in the Eastern Desert of Egypt. Am. J. Sci..

[26] Moussa H.E., Asimow P.D., Azer M.K., Abou El Maaty M.A., Akarish A.I.M., Yanni N.N., Mubarak H.S., Wilner M.J., Elsagheer M.A. (2021). Magmatic and Hydrothermal Evolution of Highly-Fractionated Rare-Metal Granites at Gabal Nuweibi, Eastern Desert, Egypt. Lithos.

[27] Awad H.A.M., Zakaly H.M.H., Nastavkin A.V., El-Taher A. (2020). Radioactive Content and Radiological Implication in Granitic Rocks by Geochemical Data and Radiophysical Factors, Central Eastern Desert, Egypt. Int. J. Environ. Anal. Chem..

[28] Hans Wedepohl K. (1995). The composition of the continental crust. Geochim. Cosmochim. Acta.

[29] Rudnick R.L., Gao S. (2014). Composition of the Continental Crust. Treatise on Geochemistry.

[30] Chiozzi P., De Felice P., Fazio A., Pasquale V., Verdoya M. (2000). Laboratory Application of NaI(Tl) γ-Ray Spectrometry to Studies of Natural Radioactivity in Geophysics. Appl. Radiat. Isot..

[31] Zakaly H.M., Uosif M.A., Madkour H., Tammam M., Issa S., Elsaman R., El-Taher A. (2019). Assessment of Natural Radionuclides and Heavy Metal Concentrations in Marine Sediments in View of Tourism Activities in Hurghada City, Northern Red Sea, Egypt. J. Phys. Sci..

[32] Awad H.A., Zakaly H.M.H., Nastavkin A.V., El Tohamy A.M., El-Taher A. (2021). Radioactive Mineralizations on Granitic Rocks and Silica Veins on Shear Zone of El-Missikat Area, Central Eastern Desert, Egypt. Appl. Radiat. Isot..

[33] Moghazy N.M., El-Tohamy A.M., Fawzy M.M., Awad H.A., Zakaly H.M.H., Issa S.A.M., Ene A. (2021). Natural Radioactivity, Radiological Hazard and Petrographical Studies on Aswan Granites Used as Building Materials in Egypt. Appl. Sci..

[34] Lasheen E.S.R., Rashwan M.A., Osman H., Alamri S., Khandaker M.U., Hanfi M.Y. (2021). Radiological Hazard Evaluation of Some Egyptian Magmatic Rocks Used as Ornamental Stone: Petrography and Natural Radioactivity. Materials.

[35] Lasheen E.S.R., Azer M.K., Ene A., Abdelwahab W., Zakaly H.M.H., Awad H.A., Kawady N.A. (2022). Radiological Hazards and Natural Radionuclide Distribution in Granitic Rocks of Homrit Waggat Area, Central Eastern Desert, Egypt. Materials.

[36] Awad H.A., Zakaly H.M.H., Nastavkin A.V., El-Taher A. (2020). Radioactive content in the investigated granites by geochemical analyses and radiophysical methods around Um Taghir, Central Eastern Desert, Egypt. J. Phys. Conf. Ser..

[37] Tawfic A.F., Zakaly H.M.H., Awad H.A., Tantawy H.R., Abbasi A., Abed N.S., Mostafa M. (2021). Natural radioactivity levels and radiological implications in the high natural radiation area of Wadi El Reddah, Egypt. J. Radioanal. Nucl. Chem..

[38] Kamar M.S. (2006). Potentiality of Nuclear Elements in Seih-Sidri Area, Southwestern Sinai-Egypt. Master’s Thesis.

[39] El- Sayed A.A., El-Aassy I.E., El-Metwally A.A., Kamar M.S. (2007). Syenogranites and associated pegmatites of Seih sidri area, southwestern sinai, Egypt: Petrographical, geochemical and radioactive characteristics. Fifth Int. Conf. Geol. Afr..

[40] Kamar M.S., Salem I.A., El-Aassy I.E., El-Sayed A.A., Rezk A.A., Saleh G.M. (2021). Contribution to the Geological, Geochemical and Mineralogical Studies of Gabal Serbal Granitic Rocks, Southwestern Sinai, Egypt. Int. J. Min. Sci..

[41] European Commission (EC) (1999). Radiological Protection Principles Concerning the Natural Radioactivity of Building Materials-Radiation Protection 112.

[42] Nada A. (2003). Evaluation of Natural Radionuclides at Um-Greifat Area, Eastern Desert of Egypt. Appl. Radiat. Isot..

[43] Uosif M.A.M., El-taher A. (2008). Radiological Assessment of Abu-Tartur Phosphate, Western Desert Egypt. Radiat. Prot. Dosim..

[44] Abed N.S., Monsif M.A., Zakaly H.M.H., Awad H.A., Hessien M.M., Yap C.K. (2022). Assessing the radiological risks associated with high natural radioactivity of microgranitic rocks: A case study in a northeastern desert of Egypt. Int. J. Environ. Res. Public Health.

[45] Saudi H.A., Abedelkader H.T., Issa S.A.M., Diab H.M., Alharshan G.A., Uosif M.A.M., Bashter I.I., Ene A., Ghazaly M.E., Zakaly H.M.H. (2022). An In-Depth Examination of the Natural Radiation and Radioactive Dangers Associated with Regularly Used Medicinal Herbs. Int. J. Environ. Res. Public Health.

